# Subcutaneous Histiocytoid Sweet Syndrome Associated with Crohn Disease in an Adolescent

**DOI:** 10.1155/2014/954254

**Published:** 2014-03-26

**Authors:** Rosa María Fernández-Torres, Susana Castro, Ana Moreno, Roberto Álvarez, Eduardo Fonseca

**Affiliations:** ^1^Department of Dermatology, University Hospital of La Coruña, Xubias de Arriba 84, 15006 La Coruña, Spain; ^2^Department of Pediatrics, University Hospital of La Coruña, Xubias de Arriba 84, 15006 La Coruña, Spain; ^3^Department of Pathology, University Hospital of La Coruña, Xubias de Arriba 84, 15006 La Coruña, Spain

## Abstract

We report a case of subcutaneous histiocytoid Sweet syndrome in an adolescent with Crohn disease. A 14-year-old boy with a 1-year history of ileocolonic and perianal Crohn disease, treated with infliximab and azathioprine, was admitted to the Pediatrics Department with malaise, abdominal pain, bloody diarrhea, and fever (39°C) from 15 days ago. Two days later, he developed cutaneous lesions consisting of tender, erythematous, and violaceous papules and nodules scattered over his legs, soles, and upper extremities. Laboratory studies revealed neutrophilia, microcytic anemia, and elevation of both erythrocyte sedimentation rate and C-reactive protein rate. A skin biopsy specimen showed deep dermal and predominantly septal inflammatory infiltrate in the subcutaneous tissue composed of polymorphonuclears, eosinophils, and mononuclear cells of histiocytic appearance. These histiocytoid cells stained positive for myeloperoxidase. Subcutaneous Sweet syndrome is a rare subtype of acute neutrophilic dermatosis, in which the infiltrate is exclusively or predominantly located in the subcutaneous tissue, causing lobular or septal panniculitis. It is often described in patients with an underlying haematological disorder or caused by drugs, but very rare in patients with inflammatory bowel disease, especially in childhood or adolescence. To our knowledge, this is the first case of subcutaneous histiocytoid type in a paediatric patient.

## 1. Introduction

Sweet syndrome, also referred to as acute febrile neutrophilic dermatosis, is a reactive condition of unknown etiology characterized by an abrupt onset of cutaneous lesions consisting of painful, erythematous plaques, papules, and nodules accompanied by fever and neutrophilia. It has been reported in association with several drugs and inflammatory, neoplastic, and infectious diseases [1].

Cases of Sweet syndrome in patients with Crohn disease have been sporadically reported. Herein, we report a case of this association with the peculiarities being the subcutaneous histiocytoid variant of Sweet syndrome and occurring in an adolescent.

## 2. Case Presentation

**Figure 1:**
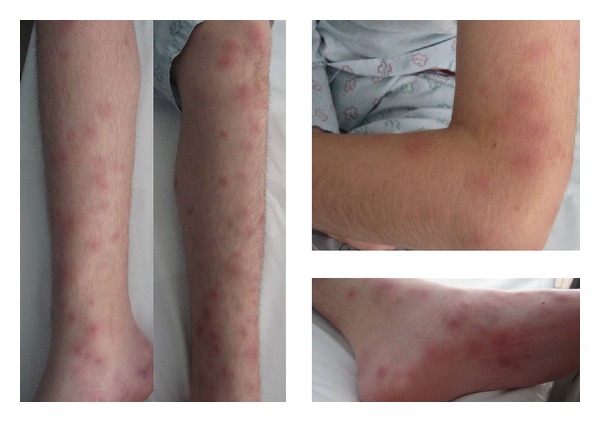
Erythematous nodules and plaques on the legs, arms, and soles.

A 14-year-old boy with a 1-year history of recurrent ileocolonic and perianal Crohn disease, treated with infliximab and azathioprine, was admitted to the Pediatrics Department with malaise, abdominal pain, bloody diarrhea, and fever (39°C) from 15 days ago. Due to suspicion of concurrent intra-abdominal infection, treatment with teicoplanin, meropenem, and metronidazole was started. Two days after hospital admission, he developed cutaneous lesions consisting of tender, erythematous, and violaceous papules and nodules scattered over his legs, soles, and upper extremities (Figure 1). Laboratory studies revealed a total white cell count of 18.85 × 10^9^ g/L with 76.4% neutrophils, microcytic anemia (serum hemoglobin: 11.20 g/dL), and elevation of both erythrocyte sedimentation rate (101 mm/hour; normal value <20 mm/hour) and C-reactive protein rate (15.30 mg/dL; normal value <1 mg/L). All other laboratory parameters were within the normal range. Blood, urine, and stool cultures were all negative as well as serology for HIV, hepatitis B, hepatitis C, and autoimmunity studies.

**Figure 2:**
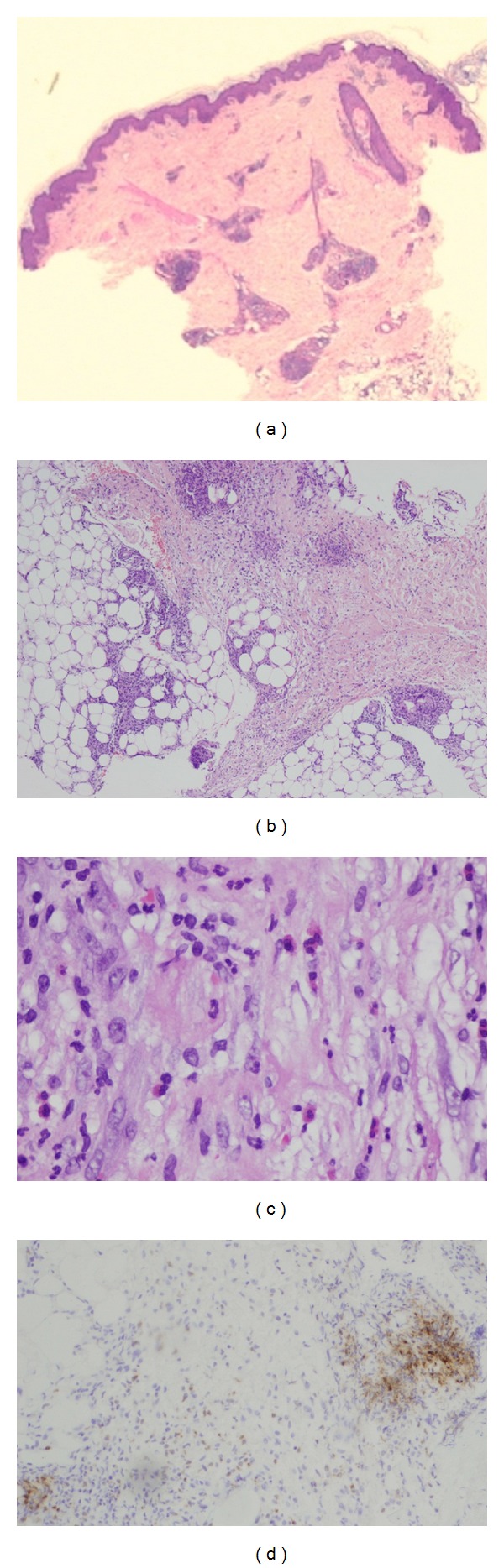
(a) Normal epidermis and dermis. Inflammatory infiltrate affecting exclusively the adipose tissue (hematoxylin-eosin, original magnification ×2). (b) Inflammatory infiltrate in the subcutaneous tissue, predominating in the septa, but also in the fat lobules (hematoxylin-eosin, original magnification ×10). (c) Infiltrate of polymorphonuclear cells, eosinophils, and large, mononuclear cells with histiocytoid appearance (hematoxylin-eosin, original magnification ×20). (d) Immunoreactivity for myeloperoxidase in most of the cells of the inflammatory infiltrate.

A skin biopsy specimen showed deep dermal and predominantly septal inflammatory infiltrate in the subcutaneous tissue composed of polymorphonuclear cells, eosinophils, and mononuclear cells of histiocytic appearance. These histiocytoid cells stained positive for myeloperoxidase (Figure 2).

Based on these findings, the diagnosis of subcutaneous histiocytoid Sweet syndrome associated with Crohn disease was established.

Methylprednisolone therapy was started at dose of 1 mg/kg daily with tapering over the next four weeks. Significant improvement was observed since the start of treatment with disappearance of fever within 24 hours and gradual involution of skin lesions.

## 3. Discussion

Sweet syndrome is a type of neutrophilic dermatosis characterized by pyrexia, elevated neutrophil counts, tender erythematous papules, nodules or plaques, and a predominantly mature neutrophilic dermal infiltrate. Subcutaneous Sweet syndrome is a rare subtype, in which the neutrophilic infiltrate is exclusively or predominantly located in the subcutaneous tissue, causing lobular or septal panniculitis [1].

In recent years, there have been several reports of a distinct variant of Sweet syndrome, the so-called histiocytoid Sweet syndrome, characterized by an infiltrate composed of histiocytoid mononuclear cells, often with elongated vesicular nuclei and ample cytoplasm, which can lead to consider histiocytic, lymphocytic, or myeloid lineages as possibilities. The most striking immunohistochemical finding is the reactivity for myeloperoxidase in most of these cells, suggesting that they are immature myeloid cells, specifically neutrophil precursors [2]. Clinically, the lesions often present as dermal nodules which may mimic erythema nodosum, especially when they are located on the legs, but can be distinguished by different histological findings.

Sweet syndrome may represent a hypersensitivity or immunological phenomenon and is known to occur in association with many drugs, paraneoplastic and inflammatory conditions as inflammatory bowel disease, haematopoietic malignancies, or solid tumours [3]. Sweet syndrome is an unusual extraintestinal manifestation of Crohn's disease. In the few cases reported, there is predilection for adult women (87%), patients with colonic disease (100%), and those with other extraintestinal manifestations (77%) [4]. Although the relationship between skin and intestinal disease is variable, cutaneous manifestations were associated with active intestinal disease in 67–80% of cases but may precede the onset of intestinal symptoms in 21% [4].

We report a case of subcutaneous histiocytoid Sweet syndrome, a rare histopathological variant often described in patients with an underlying haematological disorder or caused by several drugs, but very rare in patients with inflammatory bowel disease [5, 6].

Our patient was an adolescent with Crohn's disease receiving pharmacological treatment. Although Sweet syndrome has been associated with certain drugs as azathioprine [7], in this case, skin lesions completely cleared despite continuing with all of the drugs.

Treatment is the same as in classic Sweet syndrome, with an excellent response to oral corticosteroids, alone or in combination with metronidazole, acting synergistically.

## 4. Conclusion

We report a case of histiocytoid Sweet syndrome with the distinct features of being the subcutaneous variant occurring in an adolescent with Crohn's disease. To our knowledge, this is the first case of subcutaneous histiocytoid Sweet syndrome in a paediatric patient.
